# PIPEBAR and OverlapPER: tools for a fast and accurate DNA barcoding analysis and paired-end assembly

**DOI:** 10.1186/s12859-018-2307-y

**Published:** 2018-08-08

**Authors:** Renato Renison Moreira Oliveira, Gisele Lopes Nunes, Talvâne Glauber Lopes de Lima, Guilherme Oliveira, Ronnie Alves

**Affiliations:** 1 Instituto Tecnológico Vale, Belém, Pará Brazil; 2 Computer Science Graduate Program (PPGCC), UFPA (Pará-PA), Belém, Pará Brazil; 3 Laboratory of Bioinformatics and High-performance Computing (LaBioCAD), UFPA (Pará-PA), Belém, Pará Brazil; 4 Genetics Graduate Program, UFPA (Pará-PA), Belém, Pará Brazil

**Keywords:** Sanger, DNA barcode, DNA sequencing, Paired-end assembly

## Abstract

**Background:**

Taxonomic identification of plants and insects is a hard process that demands expert taxonomists and time, and it’s often difficult to distinguish on morphology only. DNA barcodes allow a rapid species discovery and identification and have been widely used for taxonomic identification by targeting known gene regions that permit to discriminate these species. DNA barcode sequence analysis is usually carried out with processes and tools that still demand a high interaction with the user or researcher. To reduce at most such interaction, we proposed PIPEBAR, a pipeline for DNA chromatograms analysis of Sanger platform sequencing, ensuring high quality consensus sequences along with efficient running time. We also proposed a paired-end reads assembly tool, OverlapPER, which is used in sequence or independently of PIPEBAR.

**Results:**

PIPEBAR is a command line tool to automatize the processing of large number of trace files. It is accurate as the proprietary Geneious tool and faster than most popular software for barcoding analysis. It is 7 times faster than Geneious and 14 times faster than SeqTrace for processing hundreds of barcoding sequences. OverlapPER is a novel tool for overlapping paired-end reads accurately that accepts both substitution and indel errors and returns both overlapped and non-overlapped regions between a pair of reads. OverlapPER obtained the best results compared to currently used tools when merging 1,000,000 simulated paired-end reads.

**Conclusions:**

PIPEBAR and OverlapPER run on most operating systems and are freely available, along with supporting code and documentation, at https://sourceforge.net/projects/PIPEBAR/ and https://sourceforge.net/projects/overlapper-reads/.

**Electronic supplementary material:**

The online version of this article (10.1186/s12859-018-2307-y) contains supplementary material, which is available to authorized users.

## Background

Advances in DNA sequencing approaches have produced an overwhelming volume of data, followed by new data analysis software and pipelines. Next generation sequencing (NGS) platforms have been used on a wide variety of omics studies for biodiversity assessment but the traditional Sanger method is still broadly used, including genetic testing [1] and DNA barcode generation [2–4]. DNA barcoding is an important molecular methodology based in a short standardized polymorphic sequence capable of distinguishing species. This approach is widely employed in biodiversity studies to identify and classify the diversity of well-known species or unexplored groups [4–6], evaluate inter- and intra-species variations [6–8], detect cryptic species or join genetically similar but morphologically distinct species [5, 7–9]. Also, DNA barcoding has been proposed for forensic identification and development of DNA reference library, since the lack of a reliable DNA barcoding reference library is the main barrier to its application [2] Sanger technology has been widely used for aiding morphological species identification because is a useful tool for identifying genetically distinct units worthy of more intense taxonomic study [3, 7–9] and creation of reference database, such as BOLD [10]. In these cases, there are necessity of sequencing of individual specimens using genome regions in order to infer evolutionary differences and identification only [5].

The universal barcode locus used for discriminating animal species is the 5′ region of the mitochondrial cytochrome c oxidase I (COI) gene [8]. For plants, the markers of choice are the large subunit of RuBisCo (rbcL) and maturase (matK) adopted as standards, but other markers are also used [11, 12]. The combination of coding genes (matK, rbcL, rpoB, ycf1 and rpoC1), noncoding spacers (atpF–atpH, trnH–psbA, and psbK–psbI) and the nuclear-encoded ribosomal internal transcribed spacer (ITS2) is highly recommended to obtain an adequate species discrimination for plants [13–16]. The success of DNA barcoding for evolutionary studies depends on an accurate selection of theses molecular markers, once distinct species group show distinct speciating taxa, retention of ancestral polymorphism and hybridization [17]. Therefore, Bioinformatics plays a key role in supporting and consolidating DNA barcoding efforts, from choosing the PCR primers to evaluating sequence quality and subsequent data analysis [18].

Several visualization tools are available for Sanger sequencing (4Peaks v1.8 [19], Chromas v2. 6.5 [20], Finch TV v1.5.0 [21], GLASS v0.4.3 [22], Geneious R11, CLCBio v11 [23], bioedit [24], SeqTrace v0.9.0 [25] and Sequencher v5.4. 6 [26]), however, few are intended for DNA barcode analysis (quality check, filtering, reads overlapping and format conversion). SPIDER [27], ClinQC [28], and SeqTrace were developed for Sanger sequenced analyses and are freely available.. SPIDER was developed exclusively for downstream analysis, so it cannot assemble barcoding sequences. It allows the calculation of both standard summary statistics (number of species, number of individuals, number of haplotypes per species, lengths of sequences, the proportion of missing data) and tests of DNA barcode data (barcode gap). ClinQC is a workflow developed in Python indicated for pre-processing, quality control and format conversion for Sanger and NGS data, however, it does not generate the consensus sequence from matching forward and reverse sequencing reads. SeqTrace allows the execution of all DNA barcode analysis steps, although the batch processing of several trace files cannot be executed in a concise command line (aka shell mode) and many mismatches and gaps are generated in building the consensus sequences. Geneious, CLCBio and Sequencher are proprietary softwares able to perform many types of analysis, from sequence alignment and assembly to phylogenetic trees generation, and can also be used to analyze Sanger DNA barcode sequences, but they are commercial softwares and they also cannot handle batch processing from the command line.

Here we introduce PIPEBAR, an automated, fast and accurate pipeline for Sanger sequenced DNA barcode data analysis. PIPEBAR wraps other freely available software, converting ABI files to fastq files ensuring a correct base call and a good quality content; it includes an additional step for high accuracy assembly of the paired reads (forward and reverse) based in a new assembly method, OverlapPER, that merges overlapping paired-end reads considering both indels and substitutions, returning both the overlapped and non-overlapped regions. We also make available an additional step for stop-codons and frameshift corrections for the final sequences assembled by PIPEBAR that are originated from coding regions, facilitating the submission of such sequences to barcode databases, such as BOLD and NCBI [29]. All these steps can be executed using one single command line, facilitating the batch-processing of many trace files. Batch processes using more than 800 sequence trace files on a single execution was faster in our pipeline than any other tool in our benchmark comparison. We used the commercial software Geneious (Version R10) as a reference tool for the comparison analysis.

The further sessions of the paper are organized as it follows: Implementation, where we show how PIPEBAR and OverlapPER operate; Results and Discussion, where we tested both PIPEBAR and OverlapPER and showed the obtained results, along with its discussion; and Conclusion, where we summarize the tools presented and how important they can be to the scientific community.

## Implementation

In the following sections, we show how both PIPEBAR and OverlapPER were implemented.

### PIPEBAR pipeline

**Fig. 1:**
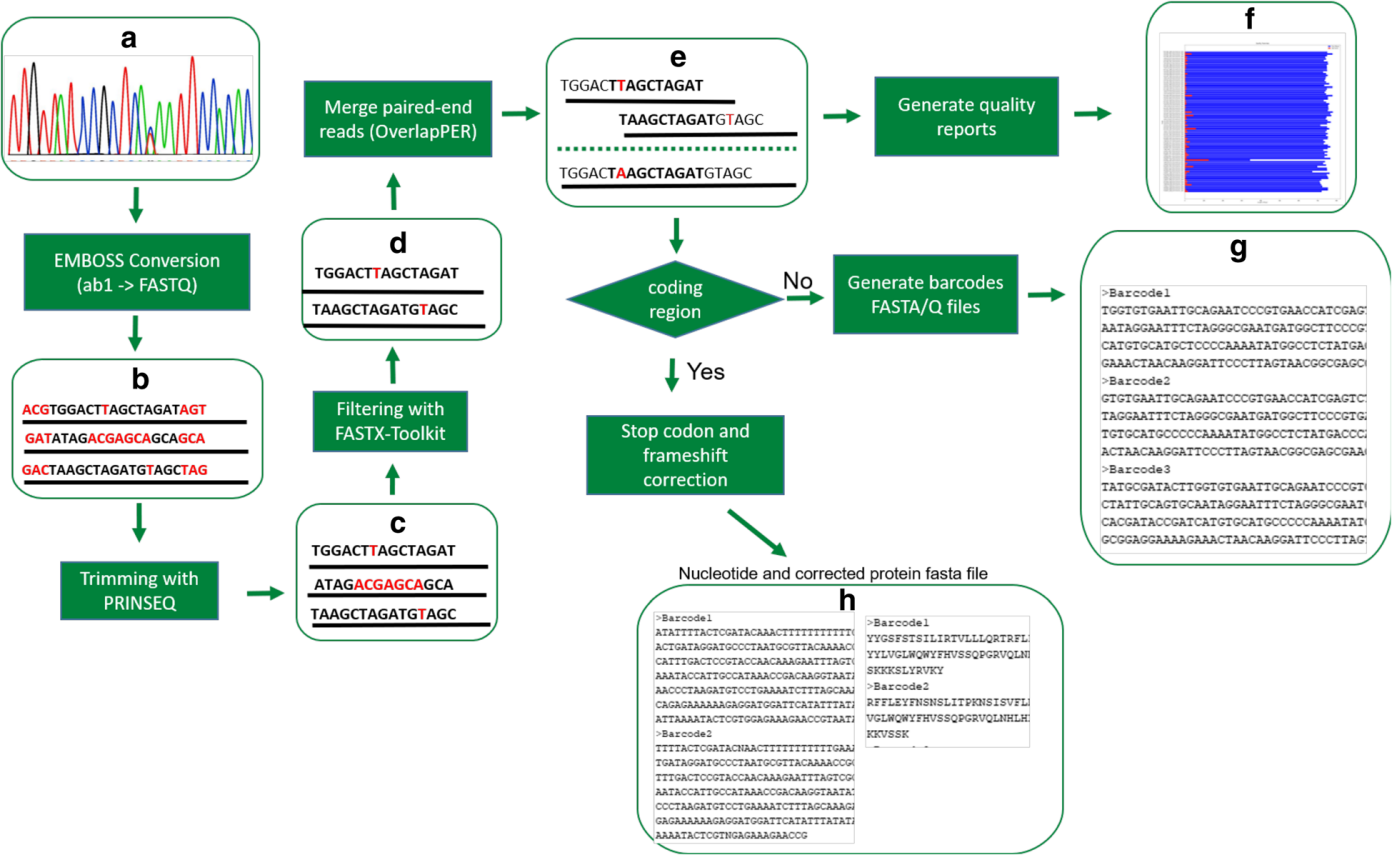
PIPEBAR workflow. **a**, **b** Conversion of chromatograms files to FASTQ files. **c**, ) Trimming and filtering of the FASTQ files for low quality bases based on Phred quality. **e** Overlapping paired-end reads considering both substitution and indel errors by OverlapPER. **f** report file of barcode sequences produced given a PHRED quality value as parameter. **g** Fasta files for the merged reads and FASTQ files for the not-merged reads. **h** In case of analyzing a coding region, PIPEBAR applies stop codon and frameshift correction

PIPEBAR was developed in a shell script for Unix-based operating systems (Linux and iOS) that organizes all the fundamental steps for obtaining high-quality barcode sequences. Figure 1 shows the PIPEBAR workflow, and all the steps will be described in detail.

At the end of the Sanger sequencing process, the output files are the ab1 and phd.1 file formats, also known as chromatograms (Fig. 1a). The initial step is converting the ab1 files to FASTQ files carried out using the EMBOSS framework [30]. The FASTQ files contain all the DNA sequences from the submitted samples along with their respective base qualities that need to be evaluated and considered in other downstream processes. After the conversion (Fig. 1b), the FASTQ files are submitted to trimming (Fig. 1c) and filtering (Fig. 1d), given a set of quality parameters (default is: minimum quality score = 20), using PRINSEQ [31]. The trimming process will discard both 5′ and 3′ ending if their mean quality score is below PHRED 20, given a sliding window(−trim_qual_window parameter) of 10 basepairs at a step size (−trim_qual_step) of 1 basepair. In the filtering process, PRINSEQ will discard all the sequences whose mean quality score (−min_qual_mean) is below PHRED 20 and length is less than 50 bp. We assemble the resulting high quality pair of sequences using an in-house developed python script, named OverlapPER, that merges the forward and reverse sequences, given a minimum overlap length (default is 25 bp), similarity (default is 90%) and gap size (default is maximum of 5 gaps opening) (Fig. 1e).

PIPEBAR generates as the final output a fasta file containing all the high-quality consensus sequences that passed the quality treatment and fulfilled the conditions of minimum overlap length and minimum overlap similarity to be merged (Fig. 1g). Sequences that do not pass through the quality evaluation as well as those that could not be merged are not discarded. They are kept in other files for possible further curation and inspection. PIPEBAR also generates quality reports (Fig. 1 and in Additional file 1: Figure S3), allowing visualization of the quality values of the barcode sequences bases by specifying a threshold of the minimum accepted PHRED value. With this report, the user will be able to check if there is some potential problematic barcodes and, if re-sequencing is needed.

When it comes to barcode analysis, usually the submission of the obtained barcodes in public databases, such as BOLD, is needed. The submission of barcode data is made with nucleotide and, in case of a coding region, protein sequences. To facilitate this submission, when the barcode is originated from a coding region, downstream analyses using the fasta files were implemented to evaluate stop codons and frameshifts that might happen in the protein translation. For this step, a python script was developed to detect the stop codons and execute correction steps based on the reading six frames of translation to aminoacids. The frameshift correction will be performed on the FASTA file output if the user specifies that the barcodes are from coding regions, otherwise the translation, frameshift and stop-codon correction are not needed (Fig. 1h).

**Fig. 2:**
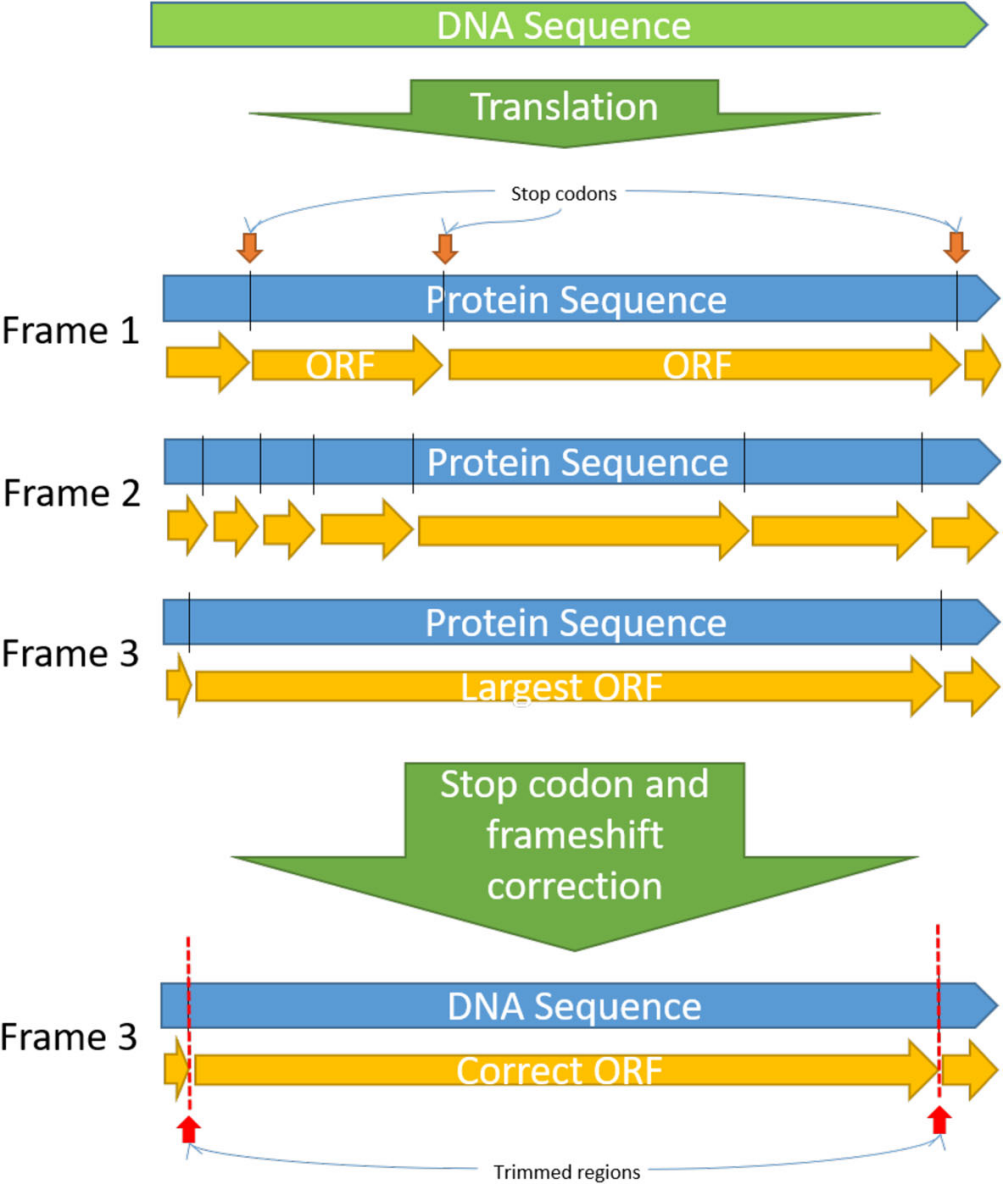
Stop-codon and frameshift corrections. PIPEBAR translates the sequence in 3 forward and 3 reverse frames, selects the frame where the impact of the found stop codons is minimum. Identifying the best translation frame, the stop codons located in the extremities of the sequence are trimmed, generating at the end of the process a sequence that is ready to be submitted to NCBI and BOLD databases

In Fig. 2 we demonstrate how the correction of stop codons and frameshifts works on PIPEBAR when the barcode is originated from a coding region. When the final output is generated, PIPEBAR will at first certify that the sequence are in the forward sense (comparing with the forward read given as input) and then will translate the nucleotide sequences to protein, according to the translation table specified by the user (default is translation Table 1 (standard)). PIPEBAR considers all the frames of translation and will choose the frame which contains the largest ORF. The chosen frame will have the 5′ and 3′ regions trimmed where the stop codons appears, resulting in the corrected ORF and respective DNA sequence.

**Table 1:** Comparison of PIPEBAR to SeqTrace and Geneious regarding to the total of barcodes produced at the end of the pipeline execution for the 3 datasets, mean similarity percentage of all the resulting barcodes to its respective Bold reference sequence, the time spent for each pipeline counting from sequences trimming to the final results, total sum of mismatches and gap openings by applying Blastn [44] against the FASTA of ab1 files retrieved from Bold

	Dataset 1 (841 plant marker genes)			Dataset 2 (558 animal marker genes)			Dataset 3 (490 fungi marker gene)		
	PIPEBAR	SeqTrace	Geneious	PIPEBAR	SeqTrace	Geneious	PIPEBAR	SeqTrace	Geneious
Resulting barcodes	830	841	829	557	558	555	448	487	438
Mean % identity	99.88 ± 0.17	99.68 ± 0.41	99.92 ± 0. 12	99.88 ± 0.16	99.56 ± 0.44	99.91 ± 0.11	99.67 ± 0.52	98.79 ± 1.73	99.73 ± 0.43
Mean % length	557.51 ± 49.2	575 ± 161. 6	549.81 ± 42.5	637.82 ± 28.98	638.54 ± 29.36	638.45 ± 28.85	618.50 ± 48.01	585.52 ± 81.53	619 ± 45.3
Run time (s)	**25**	367	197	**21**	296	98	**17**	231	160
Mismatches	372	941	294	140	383	96	267	930	224
Gap openings	91	266	41	17	115	12	82	341	72

PIPEBAR is an easy-to-use pipeline and can be used by bioinformaticians and biologists in two different ways: through a Docker environment, where the user only needs to download the PIPEBAR Docker environment (see Additional file 1) without worrying about its dependencies. The Docker environment will facilitate the use of PIPEBAR for those who do not want to install all the tools that are necessary for PIPEBAR to run properly. It is also possible to install all the dependencies of PIPEBAR separately, as shown in the Additional file 1.

### OverlapPER assembler

There are plenty of open-source tools for merging overlapping paired-end reads. and BBMerge v38.01 [32] and FLASH v1.2.11 [33] merges reads admitting substitution errors, but doesn’t handle indels. leeHom [34] also accepts only substitution error, returning only the overlapped region while merging two sequences, discarding the extremities. COPE v1.1.2 [35] and PEAR v0.9.8 [36] consider both indels and substitution errors in the assembly. However, COPE requires the kmer frequency of reads to consider indels, while PEAR trims the 5′ and 3′ sequence extremities. PANDAseq [37] is recommended to be used on Illumina sequences, demanding even that the identifier of the reads in the FASTQ file be in the Illumina format. Geneious R10 merges paired-end reads considering both indels and substitution errors, returning both overlapped and non-overlapped sequence regions, but it requires a significant amount of RAM and CPU power and it is a commercial software. To cover the gaps indicated we created OverlapPER, an open source tool that merges overlapping paired-end reads considering both indels and substitutions, returning both the overlapped and non-overlapped regions. OverlapPER may be used in sequences originated from any sequencing platform, demanding only that the pair of sequences do overlap.

**Fig. 3:**
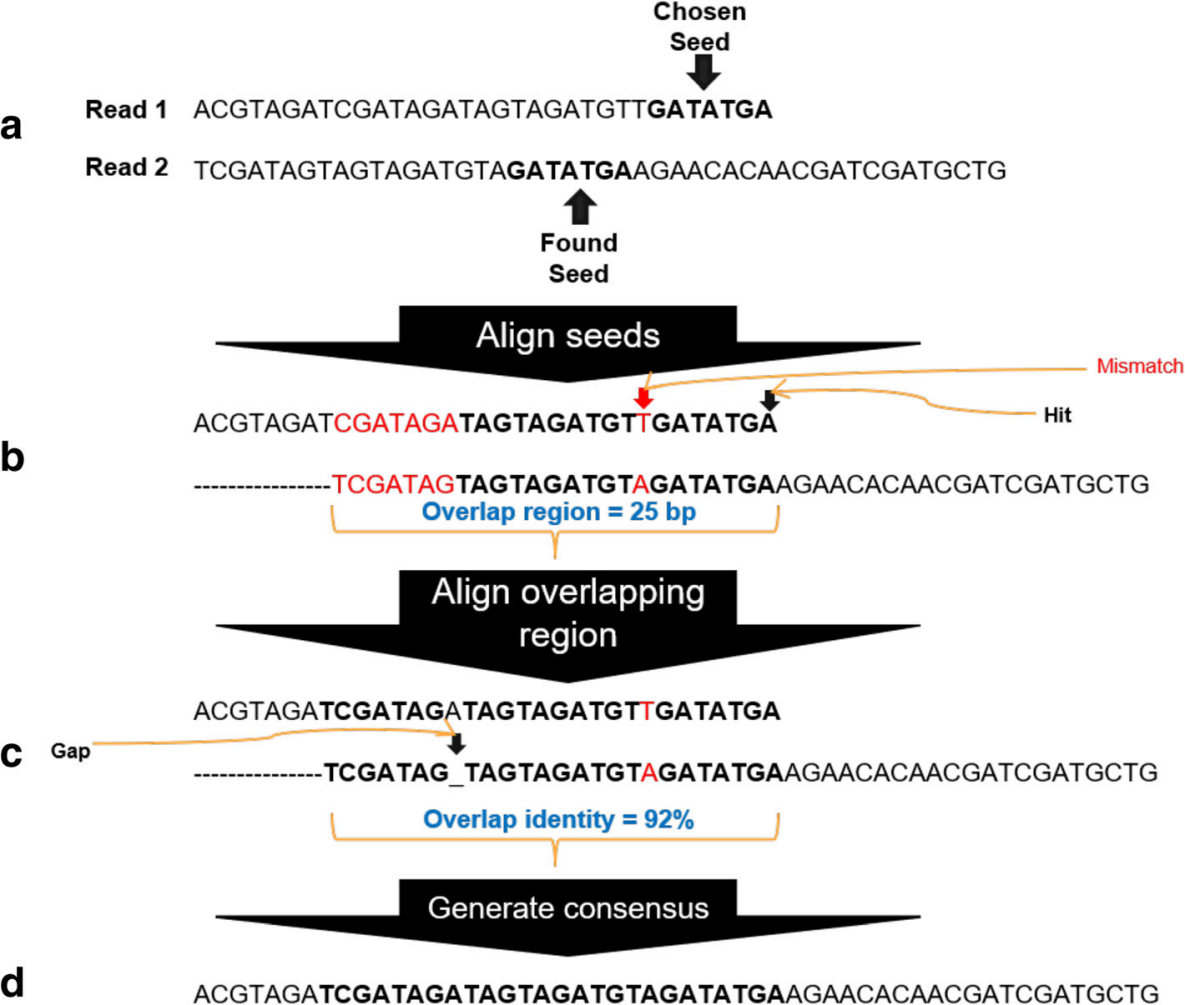
Merging process for paired-end reads. **a** OverlapPER script first finds a seed (a short sequence in one of the reads represented in bold) (**b**) The reads are positioned according to the seed found and the total overlap is determined. **c** The total overlap is analyzed. If there is a hit in the alignment, the identity score is incremented. If a base is aligned to a gap, the identity score is incremented. In case of a mismatch in the alignment, if the next 5 bases (tolerance) are identical, the mismatch score is incremented, otherwise a gap insertion is repeated 4 times until the next 5 bases are identical. Nucleotides in bold represent a hit in the alignment

OverlapPER was implemented in Python and can be executed in any operational system supporting Python 3+. It is a script for building the consensus sequences considering indels by inserting a gap in case of a mismatch and evaluates if the insertion optimizes the alignment. In case of a true mismatch, it chooses the base that has the higher quality value. OverlapPER requires as input two fastq files, containing the forward and reverse sequences in the same order in both files. Minimum accepted overlap length and the minimum similarity percentage of the overlapped region are obligatory parameters to run OverlapPER. Figure 3 exemplifies the merging of two reads (Fig. 3a) and the result (Fig. 3c). In this example, there were 8 initial mismatches (Fig. 3b, nucleotides in red) and the result obtained by OverlapPER contained only 1 mismatch and 1 gap opening (Fig. 3c, gap opening represented as a “_”).

If a generic base (N) is found in a overlapping region, OverlapPER will consider the base of the other sequence, otherwise, it will keep the generic base.

The overall algorithm of OverlapPER is as follows:

1. Finds a seed at the end of the first read (Fig. 3a);
2. Repeat if a seed is not shared between the read-pairs:
  2.1 Another seed is found in the first read, by sliding the seed window given a *seed_step* parameter;
3. The total overlap is determined considering the seed shared between the reads (Fig. 3b);
4. Repeat until all the bases from the total overlap are analyzed (Fig. 3c):
  4.1 If there is a hit in the alignment, the identity score is incremented;
  4.2 If a base is aligned to a gap, the identity score is incremented;
  4.3 If there is a mismatch in the alignment:
    4.3.1 If the next 5 bases (*tolerance*) are identical, the mismatch score is incremented;
    4.3.2 Else, repeat gap openings 4 times (*repeat*), until the next 5 bases are identical.
5. If the overlap length and the identity percentage fulfill the minimum requisites, the read-pairs are merged, generating a consensus sequence (Fig. 3d)

*Seed_length*, *seed_step*, *tolerance* and *repeat* parameters can be configured and have their default values equals to 16 bp, 12 bp, 5 bp and 4, respectively. Seminal works on the problem of deriving the consensus sequence are in [38–40].

OverlapPER is also available for use independently of PIPEBAR assuming that the intention will be only to merge paired-end reads (https://sourceforge.net/projects/overlapper-reads/).

## Results and discussion

### PIPEBAR results

To evaluate the performance and efficiency of PIPEBAR, we submitted 3 different datasets which included: a set of 436, 260 and 145 pairs of trace files (totalizing 841 pairs) corresponding to plant marker genes (rbcL, ITS2 and matK, respectively); 559 pairs of trace files for COI (animal marker gene) and another set with 490 pairs of trace file for fungal ITS. The forward and reverse trace files used as input were downloaded from the BOLD database and can be obtained at https://sourceforge.net/projects/pipebar/files/TraceFiles/. We compared the results of PIPEBAR to SeqTrace (Version 0.9.0) and Geneious (Version R10) (our benchmark), as shown in Table 1. PIPEBAR and Geneious were executed with the same parameters of minimal overlap length (25 bp), minimal similarity percentage (90%) and error probability threshold (1%). SeqTrace does not use the overlap technique to assemble its sequences, it makes use of a Bayesian inference to build the consensus sequence. For our comparison study, SeqTrace was executed with its default settings (consensus algorithm = Bayesian and a minimum confidence score = 20) which is a setting recommended by SeqTrace’s developers and authors. Table 1 shows the results obtained by running PIPEBAR, SeqTrace and Geneious in a six-core 1.9 GHz Intel Xeon computer with 32 GB of RAM, running Ubuntu 16.04 LTS.

**Fig. 4:**
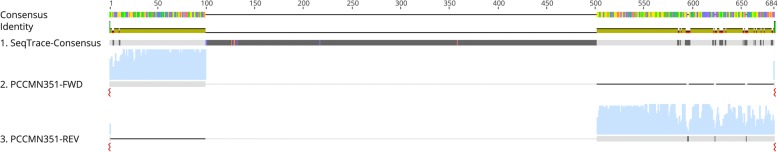
Example of an erroneous sequence generated by SeqTrace. Sequences PCCMN351-FWD and PCCMN351-REV are the trimmed sequences that should overlap, originated from the Dataset 1. SeqTrace erroneously insert gaps (from base 100 to 500) that connect the two sequences without having any insert distance information. The figure was generated using Geneious by aligning the PCCMN351-FWD and PCCMN351-REV sequences to its respective barcode generated by SeqTrace. The qualities of the forward and reverse sequences are demonstrated as a histogram. As SeqTrace does not generate a FASTQ file, we could not evaluate the quality of the barcode generated

In all the 3 datasets, PIPEBAR had the best run time results, being in average 7 times faster than Geneious and 14 times faster than SeqTrace. It also reported higher accuracy with respect to the assembled barcode sequences regarding the mean % identity when aligned to the BOLD reference sequences, being better than SeqTrace with a slightly disadvantage in comparison to Geneious (Table 1). SeqTrace obtained the highest number of resulting barcodes because it cannot consider a minimum overlap length, so it assembled pairs of sequences even if they did not share an overlapping region. Therefore, some of its resulting barcodes had a low-quality confidence (Fig. 4). Additionally, the high number of mismatches and gaps (Table 1) obtained by SeqTrace poses problems to tree-building algorithms, making sequences appear less related than they are, forcing related sequences into different clades as a high number of mismatches and gaps will directly influence biodiversity and phylogenetic downstream analysis [41].

The erroneously assembled sequences decreased the accuracy of SeqTrace’s produced barcodes. The number of mismatches and gap openings of PIPEBAR sequences were significantly better than SeqTrace sequences and slightly inferior in comparison to Geneious results. Unlike Geneious, that uses the highest signal of the chromatogram trace files in base calling process, the EMBOSS tools used by PIPEBAR calls a generic base (N) in case of discordance of the signals in a base with low quality, thus ensuring the confidence of the bases generated during basecalling.

SeqTrace obtained a mean sequence length greater than PIPEBAR and Geneious. However, upon closer inspection, one can observe that the sequences were indeed erroneously assembled sequences (Fig. 4).

To show why PIPEBAR did not assemble some sequences, we chose the results obtained from the Dataset 1. A total of 11 pairs of sequences were not assembled by PIPEBAR and Geneious. In Table 2, we show that our assembly criteria (minimum overlap length = 40 bp, minimum similarity percentage = 90% and probability limit error = 1%) were determinant to correctly build consensus over such pairs. Two of these pairs (PCCMN363 and PCCMN351) create the same problem of erroneously insertion of gaps as shown form SeqTrace (Fig. 4). Furthermore, 5 sequence pairs did not assemble as they did not overlap under either PIPEBAR nor Geneious. Two sequence pairs (PCUBC568-ITS, PCUBC799-ITS) reached the minimum overlap similarity criteria, but did not fulfill the minimum overlap length. The last 2 sequence pairs (VASCB012-ITS and VASCB062-ITS) reached the minimum overlap length criteria but they did not observed the minimum overlap similarity threshold.

**Table 2:** Overlap similarities and length of sequence pairs that were not assembled by PIPEBAR nor Geneious in the Dataset 1

Sequence ID	Overlap similarity (%)	Overlap length (bp)
BBYUK2200-ITS	–	0
MKTRT2524-rbcL	–	0
PCCMN290-ITS	–	0
PCCMN303-ITS	–	0
PCUBC495-ITS	–	0
PCUBC568-ITS	100%	20
PCUBC799-ITS	91%	12
VASCB012-ITS	27.3%	189
VASCB062-ITS	40.9%	104

The similarities were calculated by aligning the overlapping regions from each sequence pair using MAFFT [45]

The overall base quality of the final sequences obtained by both PIPEBAR and Geneious are available in the Additional file 1. As SeqTrace does not generate a FASTQ file, it was not possible to evaluate final sequence quality.

As we considered Geneious our benchmark, since it obtained the best results showed in Table 1, we also compared its results to PIPEBAR’s in all 3 datasets to see how close PIPEBAR’s results are from Geneious’, as Table 3 shows. The results indicate that PIPEBAR’s barcode sequences are almost identical to the sequences generated by Geneious toolbox, with the lowest identity percentage being 99.3% in Dataset 3.

**Table 3:** Analysis of PIPEBAR’s barcodes with respect to the mean similarity percentage of all the generated sequences to its respective Geneious’ sequences, mean length of the alignment, total sum of mismatches and gap openings by applying Blastn against the Geneious’ reference sequences

	Mean % identity	Mean length (bp)	Mismatches	Gap openings
Dataset 1	99.9 ± 0.08	545.74 ± 105.95	191	45
Dataset 2	99.97 ± 0.03	669.43 ± 19. 26	59	21
Dataset 3	99.93 ± 0. 12	596.72 ± 93.72	57	42

Additionally, we added a trusteeship step in order to facilitate the submission process of the barcode sequences to Genbank and to provide correct data for downstream analyses while handling with barcodes originated from coding regions. Stop codon and frameshifts are a problem during the sequences submission to NCBI. All sequences should be in the same frame and must not contain stop codons. Our algorithm is able to correct all the assembled sequences to the first frame in the 5′-3′ direction and trims the sequences at all the stop codons detected. Comparing to the others softwares described here, PIPEBAR is the only one providing such facility.

After stop codons trimming and frameshift corrections, the final barcode sequences can be submitted without further edition to databases such as BOLD and NCBI regarding the presence of stop codons. This is an additional step not present in the other tools that we have tested.

### OverlapPER results

To evaluate the performance of OverlapPER we used simulated data to benchmark against FLASH, COPE, BBmerge and PEAR. In our tests, IeeHom did not generate any result, even with the default parameters given in the manual, PANDAseq requires that the reads have been sequenced in Illumina Sequencers and that even the header of the reads in the FASTQ file is in Illumina format, and Geneious required a high amount of RAM, besides being a commercial software. Thus, we chose to discard these tools from comparison with OverlapPER, allowing tools that do not limit the sequence technology used to generate the data and are open-source. All the synthetic data sets are available from the OverlapPER’s website.

We used ART [42] to simulate an Illumina MiSeq v3 (2x250bp) sequencing of 1,000,000 paired-end reads from fragments with a mean size of 400 bp and a standard deviation of 10 bp, using an NCBI reference genome (*Escherichia coli* str. K-12 substr. MG1655, NC_000913.3). The dataset generated with ART can be obtained at https://sourceforge.net/projects/overlapper-reads/files/Illumina_ART_simulation/.

Table 4 shows the results obtained by running OverlapPER, FLASH, COPE and PEAR in a six-core 1.9 GHz Intel Xeon computer with 32 GB of RAM, running Ubuntu 16.04 LTS. All tools (except COPE) were with the minimum overlap of 10 bp; All tools (except PEAR) had the minimum identity percentage of the overlapped region configured to 90%. For calculating the average run time and its standard deviation, we executed each tool three times.

**Table 4:** Results obtained by OverlapPER, PEAR, FLASH and COPE

Tool	Total merged pairs	% merged pairs	Mean length of merged sequences	Mean % identity	Mean mismatch	Mean gap opening	Run time (s)
OverlapPER	999,706	99.97%	391.69 ± 18.69	97.52% ± 0.70%	7.10 ± 2.66	2.62 ± 0.9	511. 26 ± 6.87
PEAR	995,648	99.56%	391. 19 ± 20.87	96.22% ± 1.31%	11.67 ± 4.87	3.04 ± 1.18	1363.37 ± 3.22
FLASH	326,686	32.67%	391.90 ± 19.54	97.38% ± 0.73%	7.55 ± 2.87	2.71 ± 0.97	49.93 ± 2.74
COPE	292,303	29. 23%	392.34 ± 19.46	97.45 ± 0.71	7.30 ± 2.77	2.70 ± 0.97	468.45 ± 0.81
BBMerge	201,842	20.18%	392.66 ± 19.25	97.49 ± 0.70	7.03 ± 2.69	2.83 ± 0.95	25. 23 ± 0.79

Parameters: minimum overlap of 10 bp and minimum identity of 90%. Mean identity, mismatch and gap openings are shown in comparison to the reference genome

A total of 1,000,000 simulated reads were used as input for the evaluated tools. The results are shown regarding the absolute number of total merged pairs of sequences, the percentage of merged pairs, the mean length of the resulting merged sequences, the mean percentage identity when aligning the resulting sequences to the reference genome, the mean total of mismatch and gap openings resulted from the alignment and finally the mean run time took for each tool

In order to evaluate result correctness, we used BLAST+ [43] to align the merged sequences of each tool against the reference genome. OverlapPER performed best, by merging 99.97% of the paired-end reads with the highest mean similarity percentage (97.52%) and the lowest mean gap openings (2.62) (Table 4). OverlapPER presents a good tradeoff between sensitivity and scalability.

PEAR almost reached the same performance of OverlapPER when comparing the total merged pairs (4058 assembled sequences less than OverlapPER), mean length of merged sequences, mean percentage of identity (1.3% less than OverlapPER), and mean gap opening. Regarding run time, PEAR took 2.5 times longer (22.7 min) than OverlapPER (8.5 min).

With the results shown above, we felt secure about including OverlapPER in the PIPEBAR workflow.

## Conclusion

PIPEBAR was devised to efficiently assist DNA barcode analysis of sequences generated by Sanger sequencing. The chromatogram trace files or pair of forward and reverse trace files are converted into a single high quality consensus sequence. The pipeline strategically wraps several open-source software, making it possible to run barcode analysis of hundreds of sequences in a fast, accurate and concise command line (shell script). Despite the many proprietary and free software available, only SeqTrace provides a complete free and open-source toolbox. However, it is, intrinsically, a stand-alone program and many sequence analysis tasks are manually assisted, demanding a high interaction with the user. SeqTrace generated a large number of mismatches and gaps in the final consensus sequences with damaging consequences for biodiversity assessment such as phylogenetic diversity analysis.

PIPEBAR is the only program producing similarly high quality consensus sequences as accurate as the widely used proprietary Geneious, but it is faster than any software available for barcoding data analysis. Furthermore, PIPEBAR can be used to facilitate the submission of barcode sequences to databases such as BOLD and NCBI.

OverlapPER was implemented to assemble a pair of forward and reverse sequences and obtained favorable results when compared to other similar tools and it is included in PIPEBAR workflow. We recommend the use of OverlapPER in bioinformatics pipelines when paired-end reads are used for genome sequencing or re-sequencing and for the production of DNA barcodes using Sanger sequencing.

## Availability and requirements

**Project name**: PIPEBAR

**Project home page**: e.g. https://sourceforge.net/projects/PIPEBAR/

**Operating system(s)**: Platform independent

**Programming language**: Python

**Other requirements**: Docker and Python 2.7+

**License**: GNU GPL

**Any restrictions to use by non-academics**: none

**Project name**: OverlapPER

**Project home page**: e.g. https://sourceforge.net/projects/overlapper-reads/

**Operating system(s)**: Platform independent

**Programming language**: Python

**Other requirements**: Python 2.7+

**License**: GNU GPL

**Any restrictions to use by non-academics**: none

## Additional file

Additional file 1: PIPEBAR and OverlapPER’s usage. Here we show all the instructions for the installation of Pipebar and all the commands used in the tests made with Pipebar, OverlapPER and the other tools used as benchmark. (PDF 639 kb)

## Abbreviations

BOLD: Barcode of life database
CBOL: Consortium for the barcode of life
COI: Cytochrome c oxidase
CPU: Central processing unit
DNA: Desoxyribonucleic acid
LTS: Long time support
NCBI: National center for biotechnology information
NGS: Next generation sequence
RAM: Random access memory
