# Supplementary material for: PIPEBAR and OverlapPER: tools for a fast and accurate DNA barcoding analysis and paired-end assembly
Source: BMC Bioinformatics. 2018 Aug 8;19:297. doi: 10.1186/s12859-018-2307-y (PMC6083499; doi:10.1186/s12859-018-2307-y)
Supplement: Supplementary file 1 — PIPEBAR and OverlapPER’s usage. Here we show all the instructions for the installation of Pipebar and all the commands used in the tests made with Pipebar, OverlapPER and the other tools used as benchmark. (PDF 639 kb) [file 12859_2018_2307_MOESM1_ESM.pdf]

PIPEBAR and OverlapPER: tools for a fast and accurate DNA barcoding analysis and paired-end assembly

Renato Renison Moreira Oliveira[a,b,c], Gisele Lopes Nunes[a], Talvane Glauber Lopes de Lima[a], Guilherme Oliveira[a,d] , Ronnie Alves[a,b,c]

[a] Instituto Tecnológico Vale, Belém, Pará, Brazil;

[b] Computer Science Graduate Program (PPGCC), UFPA (Pará-PA) , Belém, Pará, Brazil;

[c] Laboratory of Bioinformatics and High-performance Computing (LaBioCAD), UFPA (Pará-PA), Belém, Pará, Brazil.

[d] Genetics Graduate Program, UFPA (Pará-PA), Belém, Pará, Brazil.

Corresponding authors: renato.renison@gmail.com; Ronnie.Alves@itv.org

Email addresses:

RRMO: renato.renison@gmail.com

GLN: gilopesnunes@gmail.com

TGLL: talvane.glauber@gmail.com

GO: guilherme.oliveira@itv.org

RA: ronnie.alves@itv.org

Supplementary material

Obtaining the test dataset:

The whole dataset used as input to PIPEBAR, SeqTrace and Geneious can be downloaded at https://sourceforge.net/projects/pipebar/files/TraceFiles/

PIPEBAR automatic installation

To facilitate the use of PIPEBAR by the users, we created a docker image which will enable the user to run PIPEBAR without installing its dependencies.

Installation using docker (see https://docs.docker.com)**:**

A docker image is available so the installation of all required tools are already wraped up for usage along PIPEBAR.

Step 1 – Installing Docker and wget (prerequisites)

>> sudo apt-get install docker.io

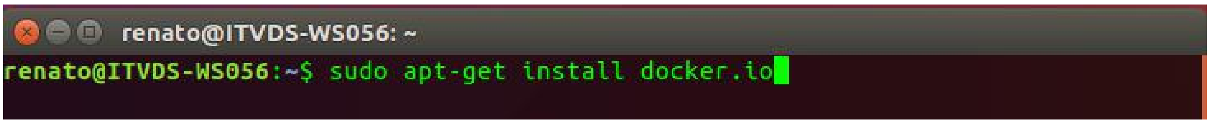

>> sudo apt-get install wget

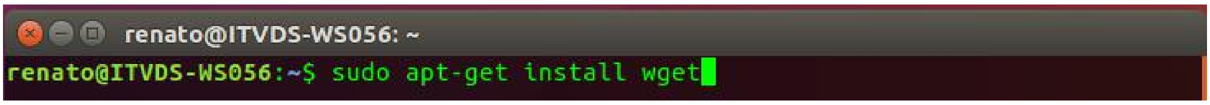

Step 2 – Checking Docker installation

>> sudo docker --version

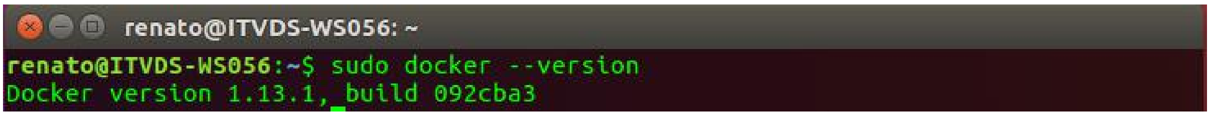

Step 3 – Downloading the Pipebar Script

In this step, you will download the script, available on SourceForge, that automatize the Pipebar pipeline. To download the script, enter:

>>wget https://sourceforge.net/projects/pipebar/files/pipebarScript.sh

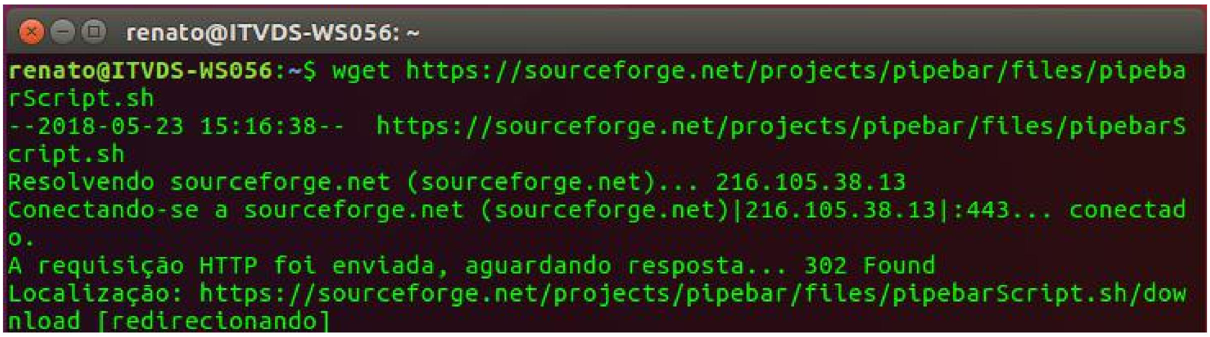

Step 4 - Initiating Pipebar

After downloading the script, you will be able to run the pipeline. With superuser permission you will type:

>>sudo sh pipebarScript.sh path/to/forward/reads path/to/reverse/reads
You need to pass two parameters, the path to forward and reverse reads. Once you entered the above command you will get a similar output, regarding the creation of the Pipebar container.

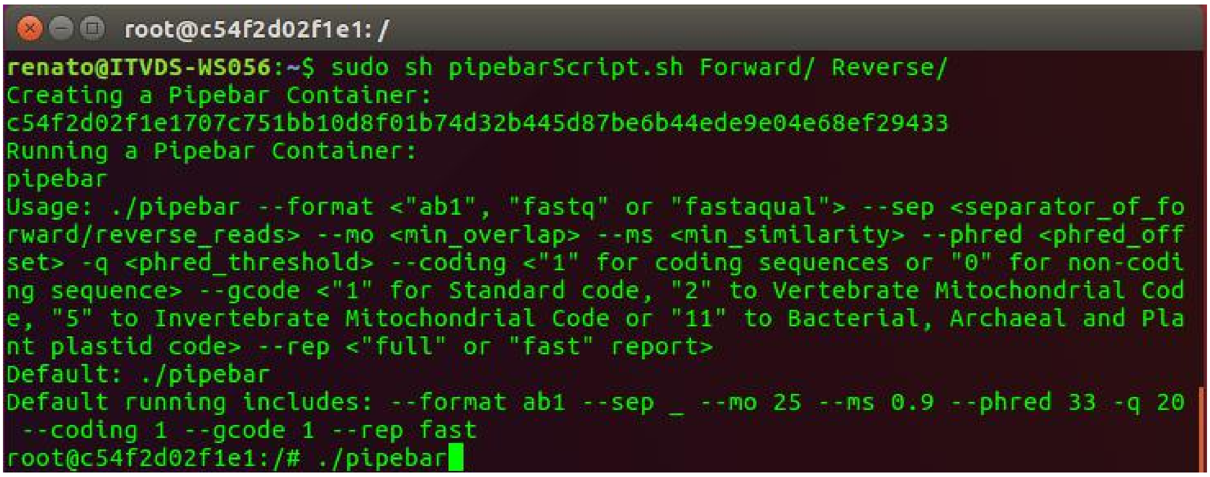

Step 5 - Running Pipebar

At this point you will be enabled to run the pipeline, as it follows.

>./pipebar --format <"ab1", "fastq" or "fastaqual"> --sep <separator_of_forward/reverse_reads> --mo <min_overlap> --ms <min_similarity> --phred <phred_offset> -q <phred_threshold> --coding <"1" for coding sequences or "0" for non-coding" sequence> --gcode <"1" for Standard code, "2" to Vertebrate Mitochondrial Code, "5" to Invertebrate Mitochondrial Code or "11" to Bacterial, Archaeal and Plant plastid code> --rep <"full" or "fast" report>

Ex: ./pipebar --format ab1 --sep _ --mo 25 --ms 0.9 --phred 33 -q 20 --coding 1 --gcode 1 --rep fast

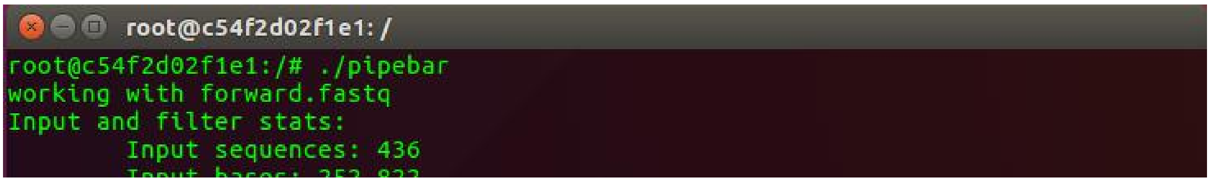

Options:

-h|--help
Show this output.
-V|--version
Show version information.
--format <string>
Input format. Can be "ab1", "fastq" or "fastaqual".
--sep <string>
The IDs from both forward and reverse reads must have a separator.
Ex: 001read_forward and 001read_reverse have "_" (default) as
separator
--mo <integer>
Length of the minimum overlap between the paired reads (default is 25).
--ms <float>
Percentage of the accepted minimum similarity in an overlap region of
two paired reads (default is 0.9).
--phred <integer>
The offset of the PHRED qualities codes used.
Can be 33 or 64 (default is 33).
-q <integer>
The minimum quality value for trimming and
filtering steps (default is 20).
--coding <integer>
Inform if the barcode sequences to be analyzed are from
coding (e.g. rbcL, matK) or non-coding (e.g. ITS, atpF-trnH) regions.
Inform "1" for coding or "0" for non-coding sequences (default is 1)
--gcode <integer>
The genetic code to be used when translating the nucleotide
sequences into protein, when it comes to a coding region. It can be
"1" to Standard Code, "2" to Vertebrate Mitochondrial Code,
"5" to Invertebrate Mitochondrial Code or "11" to Bacterial, Archaeal
and Plant plastid code.
--rep <string>
A full report will generate a quality graphical report for each
barcode sequence analyzed, while a fast report will generate an overview
of the analyzed barcodes in one single report (default is "fast")

When the pipeline finishes its execution, you need to exit the pipebar environment, just enter:
>>exit

```
root@38171f254d55:/# exit
exit
Stopping Container:
pipebar
Removing Container:
pipebar
Done!
renato@ITVDS-WS056:~$
```

Step 6 - Getting the Results

The pipebar script saves the results in the ResultPipebar folder that is in the same directory from where it was called. The resulting files are:

- notAssembled-1.fastq
- notAssembled-2.fastq
- overlaped.fasta
- overlapped.fastq
- report.pdf
- TrimmedStop_DNA.fasta
- TrimmedStop_Prot.fasta
- fastqc_report
    - overlapped_fastqc.html
    - overlapped_fastqc.zip

```
renato@ITVDS-WS056:~$ ls ResultPipebar/
fastqc_report          overlaped.fasta    TrimmedStop_DNA.fasta
notAssembled-1.fastq   overlapped.fastq   TrimmedStop_Prot.fasta
notAssembled-2.fastq   report.pdf
renato@ITVDS-WS056:~$
```

PIPEBAR manual installation

Installing dependencies manually:

You will need to download the following packages and install them:
- **EMBOSS**: ftp://emboss.open-bio.org/pub/EMBOSS/old/6.5.0/EMBOSS-6.5.7.tar.gz
- **Prinseq**: http://prinseq.sourceforge.net/
- **fastx_toolkit**: http://hannonlab.cshl.edu/fastx_toolkit/
- **BBmap**: https://sourceforge.net/projects/bbmap/?source=typ_redirect
- **FastQC**: http://www.bioinformatics.babraham.ac.uk/projects/fastqc/
- **OverlapPER**: https://sourceforge.net/projects/PIPEBAR/files/overlapper.py/download

Step 1 - Running Pipebar

At this point you will be enabled to run the pipeline, as it follows.

>./pipebar --format <"ab1", "fastq" or "fastaqual"> --sep <separator_of_forward/reverse_reads> --mo <min_overlap> --ms <min_similarity> --phred <phred_offset> -q <phred_threshold> --coding <"1" for coding sequences or "0" for non-coding" sequence> --gcode <"1" for Standard code, "2" to Vertebrate Mitochondrial Code, "5" to Invertebrate Mitochondrial Code or "11" to Bacterial, Archaeal and Plant plastid code> --rep <"full" or "fast" report>

Ex: ./pipebar --format ab1 --sep _ --mo 25 --ms 0.9 --phred 33 -q 20 --coding 1 --gcode 1 --rep fast

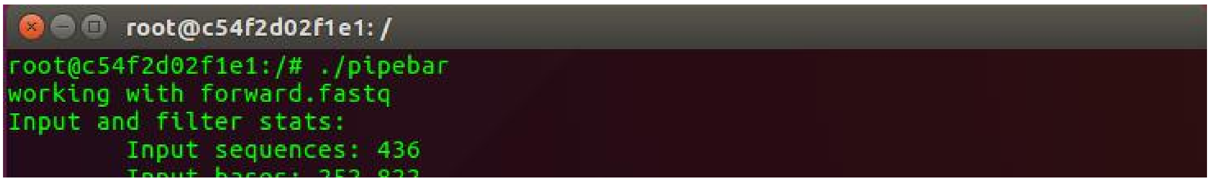

Options:

-h|--help
Show this output.

-V|--version
Show version information.

--format <string>
Input format. Can be "ab1", "fastq" or "fastaqual".

--sep <string>
The IDs from both forward and reverse reads must have a separator.
Ex: 001read_forward and 001read_reverse have "_" (default) as separator

--mo <integer>
Length of the minimum overlap between the paired reads (default is 25).

--ms <float>
Percentage of the accepted minimum similarity in an overlap region of two paired reads (default is 0.9).

--phred <integer>
The offset of the PHRED qualities codes used.
Can be 33 or 64 (default is 33).

-q <integer>
The minimum quality value for trimming and filtering steps (default is 20).

--coding <integer>
Inform if the barcode sequences to be analyzed are from coding (e.g. rbcL, matK) or non-coding (e.g. ITS, atpF-trnH) regions.
Inform "1" for coding or "0" for non-coding sequences (default is 1)

--gcode <integer>
The genetic code to be used when translating the nucleotide sequences into protein, when it comes to a coding region. It can be "1" to Standard Code, "2" to Vertebrate Mitochondrial Code, "5" to Invertebrate Mitochondrial Code or "11" to Bacterial, Archaeal and Plant plastid code.

--rep <string>
A full report will generate a quality graphical report for each barcode sequence analyzed, while a fast report will generate an overview of the analyzed barcodes in one single report (default is "fast")

Step 2 - Getting the Results

The resulting files are:

- notAssembled-1.fastq
- notAssembled-2.fastq
- overlaped.fasta
- overlapped.fastq
- report.pdf
- TrimmedStop_DNA.fasta
- TrimmedStop_Prot.fasta
- fastqc_report
    - overlapped_fastqc.html
    - overlapped_fastqc.zip

OverlapPER tests:

Simulated Dataset creation:

ART was used to simulate the Illumina sequencing of 1,000,000 paired end reads.

>art_illumina -ss MSv3 -p -sam -na -i ecoli_K-12.fasta -l 250 -c 1000000 -ir 0.09 -ir2 0.015 -dr 0.011 -dr2 0.023 -m 400 -s 10 -o illumina_ecoli_ART

The simulated data is available on https://sourceforge.net/projects/overlapper-reads/

Tools commands

OverlapPER:

>time python3 overlapper.py -f illumina_ecoli_ART1.fq -r illumina_ecoli_ART2.fq --mo 10 --ms 0.9

Merged reads: 999706

Not merged reads: 294

```
real	8m38.279s
user	8m30.844s
sys	0m7.340s
```

FLASH:

```
>time flash -t 1 -O -M 1000 -x 0.1 -o merge.assembled illumina_ecoli_ART1.fq
illumina_ecoli_ART2.fq
[FLASH] Starting FLASH v1.2.11
[FLASH] Fast Length Adjustment of SHort reads
[FLASH]
[FLASH] Input files:
[FLASH]     ../../illumina_ecoli_ART1.fq
[FLASH]     ../../illumina_ecoli_ART2-2.fq
[FLASH]
[FLASH] Output files:
[FLASH]     ./merge.assembled.extendedFrags.fastq
[FLASH]     ./merge.assembled.notCombined_1.fastq
[FLASH]     ./merge.assembled.notCombined_2.fastq
[FLASH]     ./merge.assembled.hist
[FLASH]     ./merge.assembled.histogram
[FLASH]
[FLASH] Parameters:
[FLASH]     Min overlap:           10
[FLASH]     Max overlap:           1000
[FLASH]     Max mismatch density:  0.100000
[FLASH]     Allow "outie" pairs:   true
[FLASH]     Cap mismatch quals:    false
[FLASH]     Combiner threads:      1
[FLASH]     Input format:          FASTQ, phred_offset=33
[FLASH]     Output format:         FASTQ, phred_offset=33
[FLASH]
[FLASH] Starting reader and writer threads
[FLASH] Starting 1 combiner threads
[FLASH] Processed 25000 read pairs
[FLASH] Processed 50000 read pairs
[FLASH] Processed 75000 read pairs
[FLASH] Processed 100000 read pairs
[FLASH] Processed 125000 read pairs
```

```
[FLASH] Processed 150000 read pairs
[FLASH] Processed 175000 read pairs
[FLASH] Processed 200000 read pairs
[FLASH] Processed 225000 read pairs
[FLASH] Processed 250000 read pairs
[FLASH] Processed 275000 read pairs
[FLASH] Processed 300000 read pairs
[FLASH] Processed 325000 read pairs
[FLASH] Processed 350000 read pairs
[FLASH] Processed 375000 read pairs
[FLASH] Processed 400000 read pairs
[FLASH] Processed 425000 read pairs
[FLASH] Processed 450000 read pairs
[FLASH] Processed 475000 read pairs
[FLASH] Processed 500000 read pairs
[FLASH] Processed 525000 read pairs
[FLASH] Processed 550000 read pairs
[FLASH] Processed 575000 read pairs
[FLASH] Processed 600000 read pairs
[FLASH] Processed 625000 read pairs
[FLASH] Processed 650000 read pairs
[FLASH] Processed 675000 read pairs
[FLASH] Processed 700000 read pairs
[FLASH] Processed 725000 read pairs
[FLASH] Processed 750000 read pairs
[FLASH] Processed 775000 read pairs
[FLASH] Processed 800000 read pairs
[FLASH] Processed 825000 read pairs
[FLASH] Processed 850000 read pairs
[FLASH] Processed 875000 read pairs
[FLASH] Processed 900000 read pairs
[FLASH] Processed 925000 read pairs
[FLASH] Processed 950000 read pairs
[FLASH] Processed 975000 read pairs
[FLASH] Processed 1000000 read pairs
[FLASH]
[FLASH] Read combination statistics:
[FLASH]     Total pairs:      1000000
[FLASH]     Combined pairs:   326686
[FLASH]         Innie pairs:   326626 (99.98% of combined)
[FLASH]         Outie pairs:   60 (0.02% of combined)
[FLASH]     Uncombined pairs: 673314
[FLASH]     Percent combined: 32.67%
[FLASH]
```

[FLASH] Writing histogram files.
[FLASH]
[FLASH] FLASH v1.2.11 complete!
[FLASH] 52.891 seconds elapsed

real	0m52.914s
user	0m53.356s
sys	0m3.324s

PEAR

>time pear -f illumina_ecoli_ART1.fq -r illumina_ecoli_ART2.fq -o pear.merged

```
 ____  _____    _    ____
|  _ \| ____|  / \  |  _ \
| |_) |  _|   / _ \ | |_) |
|  __/| |___ / ___ \|  _ <
|_|   |_____/_/   \_\_| \_\
```

PEAR v0.9.8 [April 9, 2015]

Citation - PEAR: a fast and accurate Illumina Paired-End reAd mergeR
Zhang et al (2014) Bioinformatics 30(5): 614-620 | doi:10.1093/bioinformatics/btt593

Forward reads file.................: illumina_ecoli_ART1.fq
Reverse reads file.................: illumina_ecoli_ART2.fq
PHRED..............................: 33
Using empirical frequencies........: YES
Statistical method.................: OES
Maximum assembly length............: 999999
Minimum assembly length............: 50
p-value............................: 0.010000
Quality score threshold (trimming).: 0
Minimum read size after trimming...: 1
Maximal ratio of uncalled bases....: 1.000000
Minimum overlap....................: 10
Scoring method.....................: Scaled score
Threads............................: 1

Allocating memory..................: 200,000,000 bytes
Computing empirical frequencies....: DONE
  A: 0.246168
  C: 0.253836
  G: 0.253816
  T: 0.246180

0 uncalled bases
Assemblying reads: 100%

Assembled reads ....: 995,648 / 1,000,000 (99.565%)
Discarded reads ....: 0 / 1,000,000 (0.000%)
Not assembled reads ....: 4,352 / 1,000,000 (0.435%)
Assembled reads file...............: pear.merged.assembled.fastq
Discarded reads file...............: pear.merged.discarded.fastq
Unassembled forward reads file.....: pear.merged.unassembled.forward.fastq
Unassembled reverse reads file.....: pear.merged.unassembled.reverse.fastq

real 22m42.668s
user 22m41.456s
sys 0m1.184s

COPE

>time cope -a illumina_ecoli_ART1.fq -b illumina_ecoli_ART2.fq -o cope_full_mode.fq -2 left1.fq -3 left2.fq -s 33 -u 1000 -c 0.9 -m 3 -t kmer-cope.freq.cz -f kmer-cope.freq.cz.len
Program start..
Program: COPE (Connecting Overlapped Pair-End reads)
Version: v1.1.2
Author: BGI-ShenZhen
CompileDate: Jul 20 2016 time: 14:35:36
Current time: Mon May 14 20:18:48 2018
Command line: cope -a ../../illumina_ecoli_ART1.fixed.fq
-b ../../illumina_ecoli_ART2-2.fq -o cope_full_mode.fq -2 left1.fq -3 left2.fq -s 33 -u 1000 -c 0.9 -m 3 -t ../../kmer-cope.freq.cz -f ../../kmer-cope.freq.cz.len
loading kmerfreq table...
kmer freq table:
kmer_num node_num error_freq_num error_ratio suspicious_freq_num
suspicious_ratio normal_freq_num normal_ratio
repeat_freq_numrepeat_ratio
468304845 94053426 88334798 0.939198 1250904 0.0132999
178635 0.00189929 4289089 0.0456027
finish loading kmer freq table!
Run time: 11s.
Begin read files and connect pairs...
Process pair reads number: 1000000
Connect reads finished!
Kmer frequency based connection table:

total_pairs	connected_pairs	connect_ratio(%)	low_quality_pairs
	low_quality_ratio(%)
1000000	292303	29.2303	0	0
starting to do cross connect for 707697 pair reads
Use connected file to cross
Count pair kmer number...
Count kmer pair number: 0
loading pair kmer information!
starting to load pair kmer file...
loading kmer file finished! Totally load kmer pair number is: 0
sort pair kmers!
load hashset!
final hash set size is 1048583
load all reads to get cross reads
Begin load read file: cope_full_mode.fq
finished load read file: cope_full_mode.fq ! totally load 292303 reads
Get cross read finished and totally cross 0 reads!
Begin to cross connect reads, processing file: cross.read.inf0.gz ...
No cross reads, cross reads connect finished!
All done!

Cross connection table:
total_pairs	no_marker	have_cross	have_cross_ratio(%)	have_cross_connect
	cross_connect_ratio(%)
0	0	0	-nan	0	-nan
All done, Thank you!
 Run time: 469s.

real	7m48.480s
user	7m28.328s
sys	0m19.956s

BBMerge:

time bbmerge.sh in1=illumina_ecoli_ART1.fixed.fq in2=illumina_ecoli_ART2-2.fq out=bbmerge_assembled.fastq outu1=bbmerge_unassembled1.fastq outu2=bbmerge_unassembled2.fastq minoverlap=10 maxratio=0.1

Version 38.01 [in1=illumina_ecoli_ART1.fixed.fq, in2=illumina_ecoli_ART2-2.fq, out=bbmerge_assembled.fastq, outu1=bbmerge_unassembled1.fastq, outu2=bbmerge_unassembled2.fastq, minoverlap=10, maxratio=0.1]

Writing mergable reads merged.
Started output threads.

Total time: 24.879 seconds.

Pairs:	1000000	
Joined:	201842	20,184%
Ambiguous:	768588	76,859%
No Solution:	29570	2,957%
Too Short:	0	0,000%

Avg Insert:	401.8
Standard Deviation:	10.1
Mode:	401

Insert range:	104 - 491
90th percentile:	415
75th percentile:	409
50th percentile:	402
25th percentile:	395
10th percentile:	389

real	0m26.408s
user	1m43.780s
sys	0m1.900s

Results validation

Blast was used in order to validate the results generated by each of the merger tools First, we created a database of the reference genome fasta file:

```
>formatdb -p F -i ecoli_K-12.fasta
```

Second, we compared all the merged sequences against the created database.

```
>blastall -p blastn -d ecoli_K-12.fasta -i ../overlapped.fastq -a 2 -m 9 -o blast_output
```

An in-house script was used to summarize all the best hits from each merged sequence.

The results of OverlapPER are also available on https://sourceforge.net/projects/overlapper-reads/

PIPEBAR results

PIPEBAR has been tested over the dataset presented above. The comparison of PIPEBAR results and the others softwares are presented in Table 1 of the main manuscript. We submitted fastq files to FastQC, in order to verify the quality of the generated sequences. Given that only PIPEBAR and Geneious generate FASTQ files as output, we only compare their results.

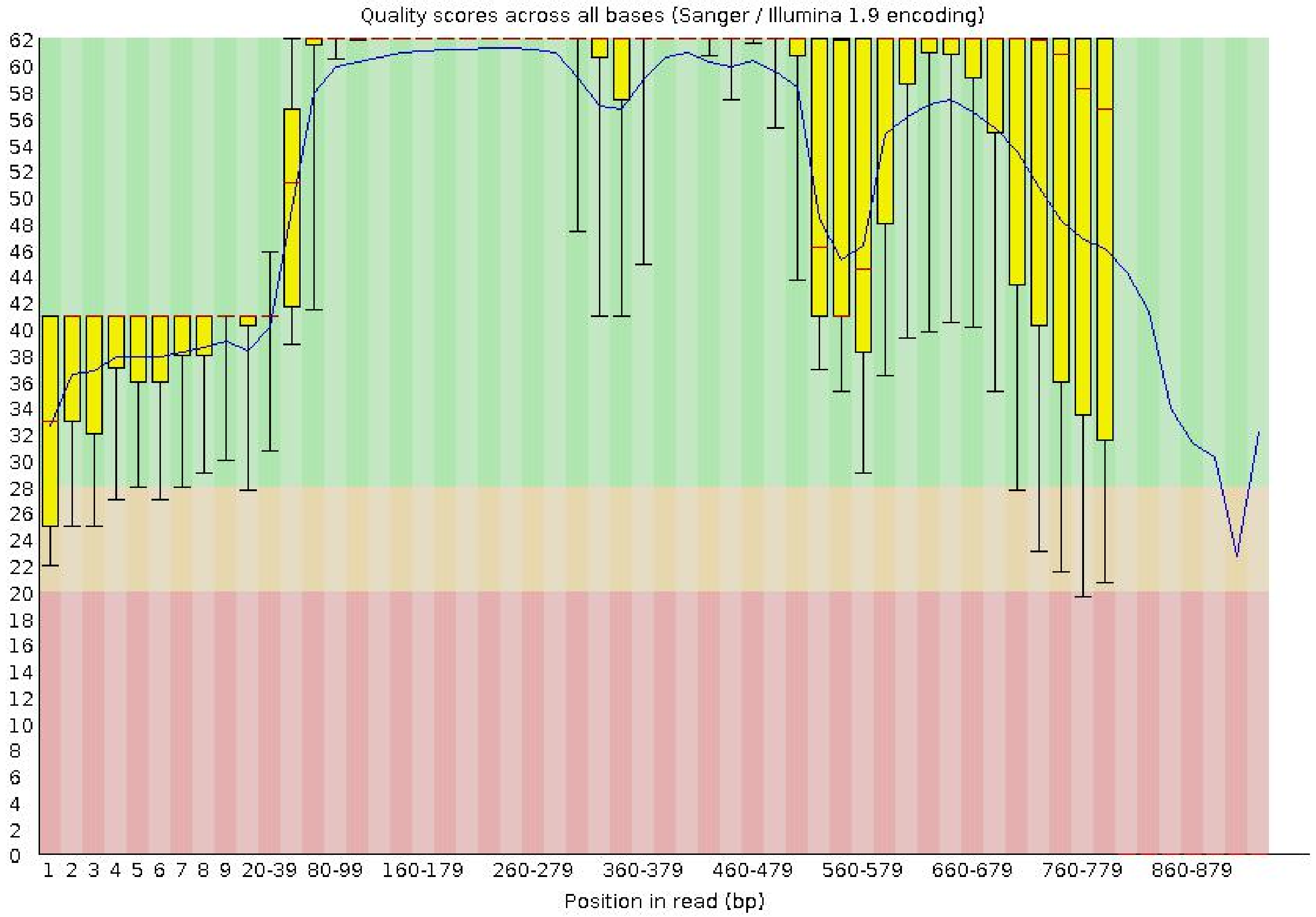

Figure S1 - Box diagram of bases quality for all sequences generated by PIPEBAR.

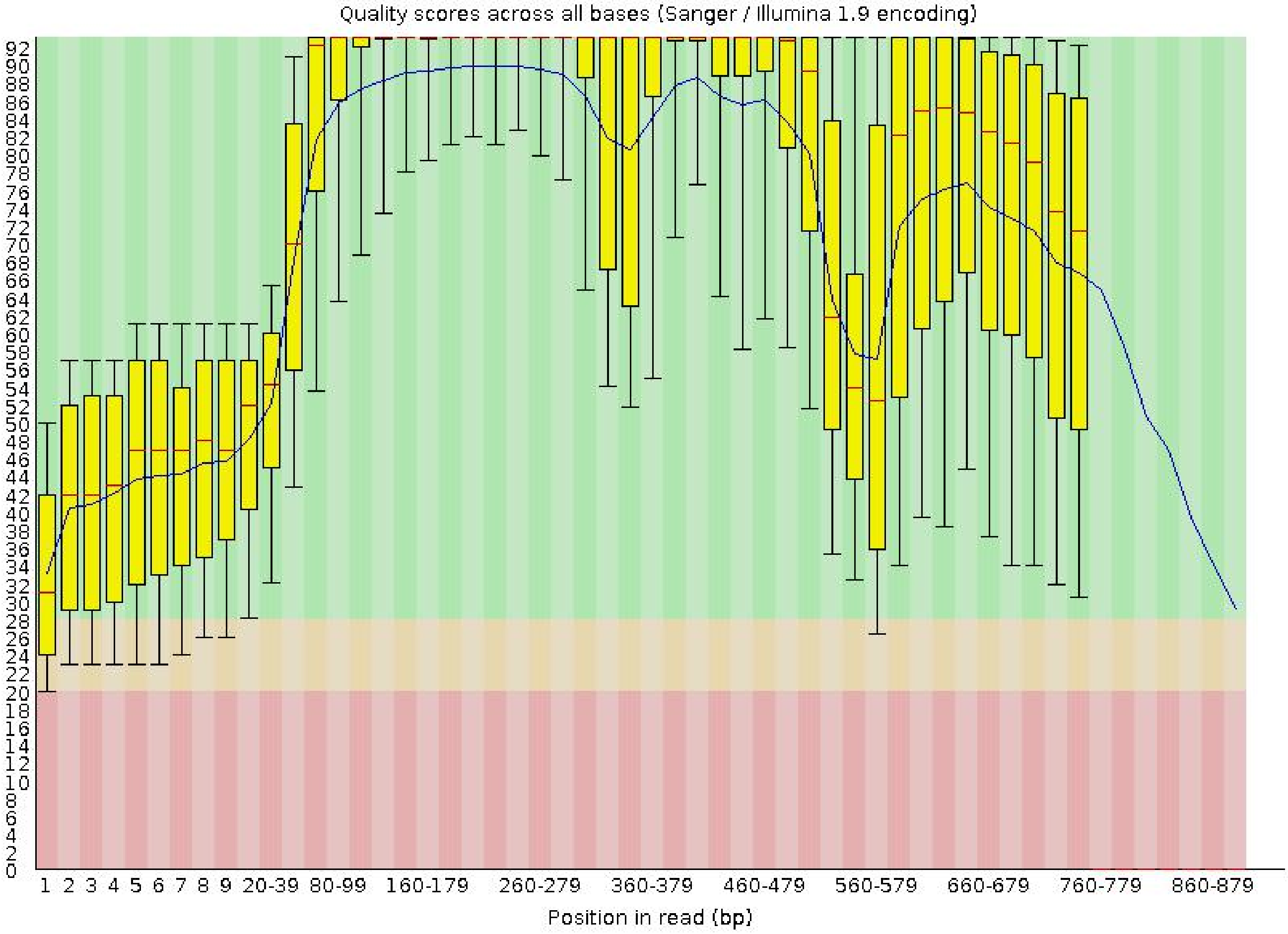

Figure S2 - Box diagram of bases quality for all sequences generated by Geneious.

Figures S1 and S2 show that the overall quality bases from the sequences generated by PIPEBAR are very similar to the quality generated by our benchmark (Geneious).

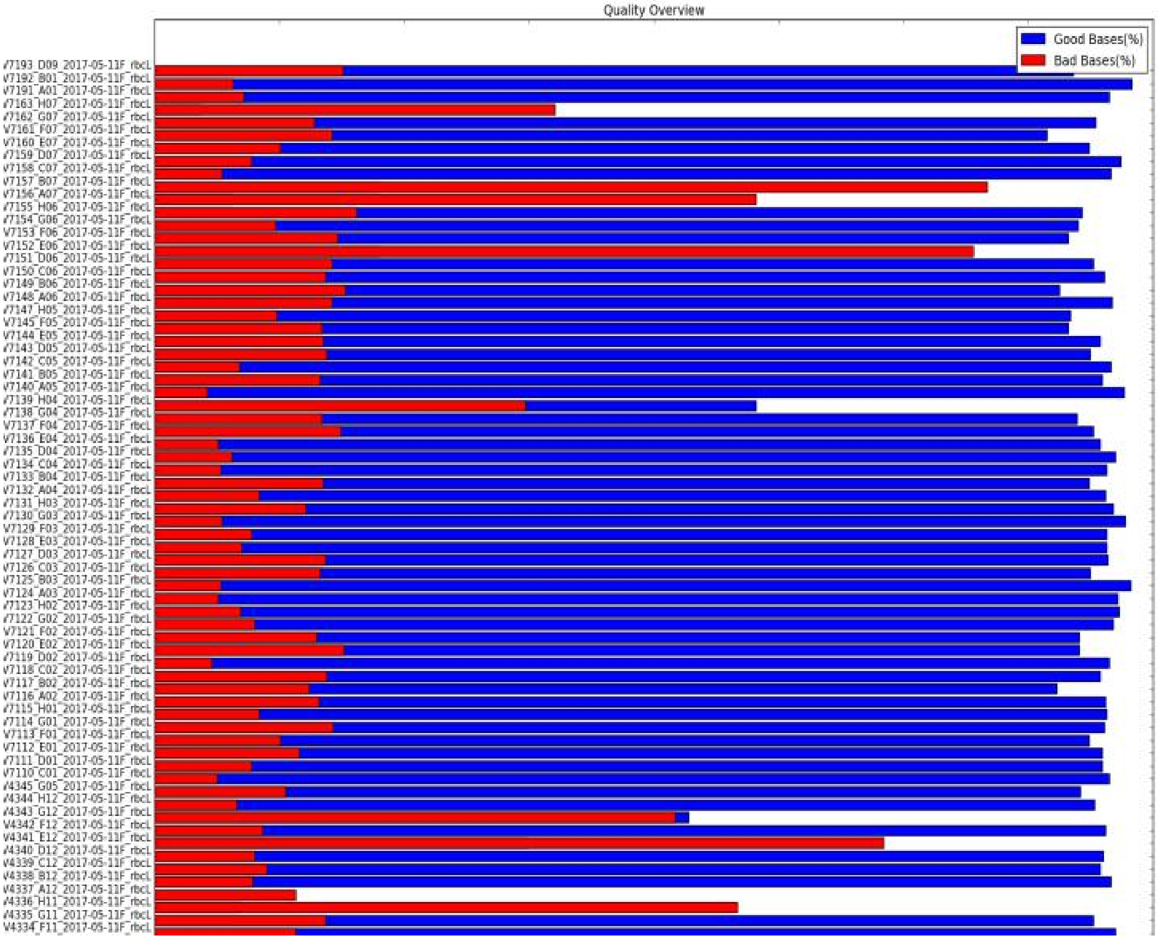

Figure S3 - Quality report generated by Pipebar. Each bar represents the quality of the bases from a barcode. The red color indicates quality below the specified PHRED threshold, while blue color indicates quality above that threshold.
